# Prognostic factors of brain metastases from colorectal cancer

**DOI:** 10.1186/s12885-019-5973-x

**Published:** 2019-07-31

**Authors:** Jun Imaizumi, Dai Shida, Yoshitaka Narita, Yasuji Miyakita, Taro Tanabe, Atsuo Takashima, Narikazu Boku, Hiroshi Igaki, Jun Itami, Yukihide Kanemitsu

**Affiliations:** 1 Department of Colorectal Surgery, National Cancer Center Hospital, 5-1-1 Tsukiji, Chuo-ku, Tokyo, 1040045 Japan; 2 Department of Neurosurgery and Neuro-Oncology, National Cancer Center Hospital, 5-1-1 Tsukiji, Chuo-ku, Tokyo, 1040045 Japan; 3 Gastrointestinal Medical Oncology Division, National Cancer Center Hospital, 5-1-1 Tsukiji, Chuo-ku, Tokyo, 1040045 Japan; 4 Department of Radiation Oncology, National Cancer Center Hospital, 5-1-1 Tsukiji, Chuo-ku, Tokyo, 1040045 Japan

**Keywords:** Brain metastases, Colorectal cancer, Karnofsky performance status

## Abstract

**Background:**

For brain metastases from non-specific primary tumors, the most frequently used and validated clinical prognostic assessment tool is Karnofsky performance status (KPS). Given the lack of prognostic factors of brain metastases from colorectal cancer (CRC) other than KPS, this study aimed to identify new prognostic factors.

**Methods:**

This retrospective cohort study was conducted at a tertiary care cancer center. Subjects were patients with brain metastases from CRC among all patients who received initial treatment for CRC at the National Cancer Center Hospital from 1997 to 2015 (*n* = 7147). Prognostic clinicopathological variables for overall survival (OS) were investigated.

**Results:**

There were 68 consecutive patients with brain metastases from CRC, corresponding to 1.0% of all patients with CRC during the study period. Median survival time was 6.8 months. One-year and 3-year OS rates were 28.0 and 10.1%, respectively. Among the six covariates tested (age, KPS, presence of extracranial metastases, control of primary lesion, number of brain metastases, and history of chemotherapy), multivariate analysis revealed KPS (score ≥ 70), number of brain metastases (1–3), and no history of chemotherapy to be independent factors associated with better prognosis.

**Conclusions:**

In addition to KPS, the number of brain lesions and history of chemotherapy were independent prognostic factors for OS in patients with brain metastases from CRC. An awareness of these factors may help gastrointestinal surgeons make appropriate choices in the treatment of these patients.

## Background

Brain metastases, the most common intracranial tumors in adults, accounts for more than half of all brain tumors [1, 2]. The incidence of brain metastases is on the rise, likely due to improved detection of small metastases by magnetic resonance imaging and improved management of extracerebral disease by progress of systemic therapy. The incidence of brain metastases by origin was reported as follows [1, 2]; lung cancer – 16 to 20%, melanoma – 7%, renal cell cancer – 7 to 10%, breast cancer – 5%, and colorectal cancer (CRC) – 1 to 2%.

Regarding prognostic factors of brain metastases, the Radiation Therapy Oncology Group (RTOG) proposed and validated recursive partitioning analysis (RPA) using Karnofsky performance status (KPS) score, which reflects a patient’s functional status [3, 4]. RPA consists of the following three classes: Class 1: patients with a KPS score ≥ 70 (score of 70: cares for self) [5] and age < 65 years with controlled primary tumor and no extracranial metastases; Class 3: KPS score < 70; and Class 2: all others [3]. KPS score, age, adequate control of primary tumor, and presence of extracranial metastases are currently considered reliable prognostic factors of brain metastases from non-specific primary cancer [3, 4, 6–9]. With respect to the establishment of criteria by origin, the diagnosis-specific graded prognostic assessment (DS-GPA), which adds the number of brain metastases to the four prognostic factors reported by RPA as covariates for multivariate analysis, was proposed and validated for non-small cell lung cancer, breast cancer, renal cell carcinoma, melanoma, and gastrointestinal cancer [10, 11]. In the DS-GPA model, KPS was the only significant prognostic factor for gastrointestinal cancer, which includes CRC, gastric cancer, and esophageal cancer [10, 12, 13].

Since survival following the treatment of brain metastases is highly variable and partly dependent on the clinical course of the primary tumor, investigating prognostic factors of brain metastases from CRC is important. While a number of reports have examined prognostic factors of brain metastases from CRC [14–18], these studies did not adopt KPS as a covariate, which as mentioned above is a significant prognostic factor in the DS-GPA model. To this end, the present study aimed to investigate prognostic factors of brain metastases from CRC, including KPS as an essential covariate, in order to provide insight that could help in the development of appropriate treatment strategies for brain metastases.

## Methods

### Patient selection and data

**Fig. 1:**
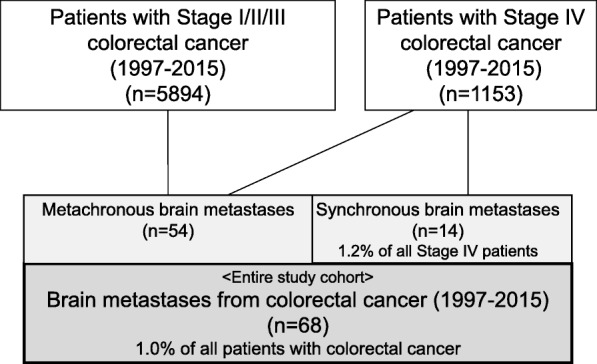
The study cohort. The final study population consisted of 68 patients with brain metastases who underwent initial treatment for colorectal cancer at the National Cancer Center Hospital from 1997 to 2015. The 68 patients account for 1.0% of all patients with colorectal cancer during the study period

The present study was retrospective in design. Inclusion criteria were patients with synchronous or metachronous brain metastases of colorectal adenocarcinoma among all patients who received initial treatment for CRC at the National Cancer Center Hospital from January 1997 to December 2015. During the study period, there were 5894 patients with Stage I/II/III CRC and 1153 patients with Stage IV CRC (Fig. 1). Targeted molecular agents were introduced in Japan for use in systemic chemotherapy for metastatic CRC during the study period (i.e., in 2007). Thus, we divided the study period into two halves (first half, 1997–2005; second half: 2006–2015).

The following parameters at the time of diagnosis of brain metastases were assessed retrospectively: sex, age, KPS score, number of brain metastases, maximum diameter of brain metastases, location of brain lesions (supratentorial or infratentorial), treatment procedure for brain metastases and parameters related to the primary tumor such as initial treatment period, time interval between diagnosis of primary tumor and brain metastases, location of primary tumor [19], presence of extracranial metastases at the time of diagnosis of brain metastases, control of primary tumor, and history of systemic chemotherapy before the development of brain metastases.

This study was approved by the Institutional Review Board (IRB) of the National Cancer Center Hospital (IRB code: 2017–437).

### Definition of Karnofsky performance status

KPS is a standard tool for measuring the ability of cancer patients to perform ordinary tasks [20]. It provides a comprehensive score that reflects a patient’s functional status, rated on a 11-point scale from 0 (death) to 100 (normal: no complaints, no evidence of disease). A higher score reflects a better ability to carry out daily activities. The cut-off identified in the RPA by the RTOG was between a score of 70 (cares for self but unable to carry on normal activity or to do active work) and 60 (requires occasional assistance but is able to care for most of personal needs).

### Treatment of brain metastases from colorectal cancer

Current treatment options for brain metastases from CRC include surgical resection, whole-brain radiotherapy (WBRT), stereotactic radiotherapy (SRT), which includes stereotactic radiosurgery and fractionated stereotactic radiotherapy, and chemotherapy, alone or in combination. In general, surgical resection is performed for a single large (typically > 3 cm) brain metastasis with massive edema or when the metastasis is located in the eloquent area. Stereotactic radiosurgery using the Gamma Knife® or CyberKnife® is usually indicated for oligometastases of small sizes up to 3 cm. In fractionated stereotactic radiotherapy, which is typically performed for larger-sized brain metastases that cannot be handled by stereotactic radiosurgery, patients are treated with the CyberKnife® using a prescribed dose of 27–35 Gy delivered in 3–5 fractions in the tumor periphery. WBRT is selected for patients with multiple metastases or large-sized oligometastases with uncontrolled extracranial metastases, and/or those with poor performance status. Typical WBRT consists of 30 Gy in 10 fractions to the isocenter, delivered five times weekly with a linear accelerator.

### Statistical analysis

Estimation of overall survival (OS) and multivariate analysis were performed as described previously [21, 22]. Briefly, OS was defined as the interval between the date of diagnosis of brain metastases and the date of death from all causes or date of last follow-up for survivors. The study cut-off date was September 2018, and patients who were alive at the end of follow-up were censored. OS was estimated by using the Kaplan-Meier method, and differences in survival were assessed with the log-rank test. Multivariate Cox proportional hazards regression models were subsequently fitted to evaluate the relationship between brain metastases from CRC and OS, while controlling for potential confounding covariates.

Data are presented as numbers of patients, ratios (%), or hazard ratios (HR) and 95% confidence intervals (CI). *P* < 0.05 was considered statistically significant. All statistical analyses were performed using the JMP14 software program (SAS Institute Japan Ltd., Tokyo, Japan).

## Results

### Characteristics of the study cohort

Details of the study cohort are summarized in Fig. 1. During the study period, 5894 patients with Stage I/II/III CRC underwent tumor resection, and 1153 patients with Stage IV CRC received treatment, for example, by tumor resection and/or systemic chemotherapy. Of the 1153 Stage IV patients, 14 (1.2% of all Stage IV patients) had synchronous brain metastases. Fifty-four patients had metachronous brain metastases. Accordingly, the final study population consisted of 68 consecutive patients (43 males and 25 females), corresponding to 1.0% of all patients with CRC during the study period (Fig. 1). The primary tumor site was the colon for 42 patients (0.9% among 4844 colon cancers) and rectum for 26 patients (1.2% among 2203 rectal cancers).

Patient characteristics are shown in Table 1. The median age at the time of brain metastases was 65 years (age range, 32–84 years). Twenty-seven patients (40%) had a KPS score ≥ 70 and 41 patients (60%) had a score ≤ 60. Fifty-four patients (79%) had extracranial disease. Eleven patients (16%) had uncontrolled primary tumors. Before the occurrence of brain metastases, 46 patients (68%) had received systemic chemotherapy. Twenty-one patients (31%) were treated with both oxaliplatin-containing and irinotecan-containing regimens. For brain metastases, 42 patients (62%) had limited (1–3) lesions, whereas 26 patients (38%) had multiple (≥4) lesions. Twenty-eight patients (41%) had brain metastases > 3 cm in maximal diameter. In terms of location of brain lesions, 39 patients (57%) had supratentorial lesions, 11 patients (16%) had infratentorial lesions, and 18 patients (27%) had both types of lesions.

**Table 1:** Patient characteristics (*n* = 68)

Characteristic	Category	No. of patients (%)
Sex	Male	43 (63%)
	Female	25 (37%)
Age at the time of brain metastases (years)	< 65	33 (49%)
	≥65	35 (51%)
Location of primary tumor	Colon	42 (62%)
	Rectum	26 (38%)
Time interval from diagnosis of primary tumor to brain metastases	Synchronous	14 (21%)
	Metachronous	54 (79%)
Karnofsky performance status score	≥70	27 (40%)
	< 70 (≤60)	41 (60%)
Presence of extracranial metastases	Absent (Brain only)	14 (21%)
	Present (Brain and other sites)	54 (79%)
Control of primary tumor	Controlled	57 (84%)
	Uncontrolled	11 (16%)
Maximum diameter of brain metastases	< 3 cm	40 (59%)
	≥3 cm	28 (41%)
Number of brain metastases	Limited: 1–3	42 (62%)
	Multiple: ≥4	26 (38%)
Interval from diagnosis of primary tumor to brain metastases	< 12 months	17 (25%)
	≥12 months	51 (75%)
Location of brain lesions	Supratentorial	39 (57%)
	Infratentorial	11 (16%)
	Both	18 (27%)
History of systemic chemotherapy before diagnosis of brain metastases	None	22 (32%)
	Fluoropyrimidine only	13 (19%)
	Fluoropyrimidine + oxaliplatin	6 (9%)
	Fluoropyrimidine + irinotecan	6 (9%)
	Fluoropyrimidine + oxaliplatin + irinotecan	21 (31%)

### Long-term outcomes after diagnosis of brain metastases and causes of death

**Fig. 2:**
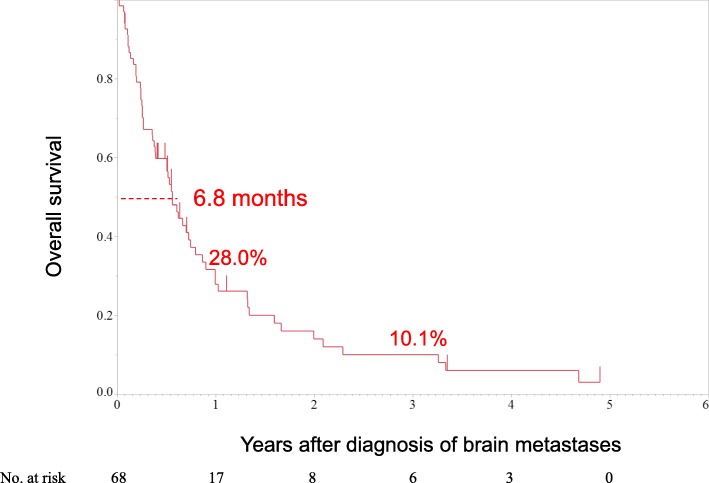
Overall survival curve of patients with brain metastases from colorectal cancer (*n* = 68). Median survival time was 6.8 months. One-year and 3-year overall survival rates were 28.0 and 10.1%, respectively

Median survival time was 6.8 months from the diagnosis of brain metastases. One-year and 3-year OS rates were 28.0 and 10.1%, respectively. Notably, no patient survived for more than 5 years (Fig. 2). During the study period, 57 of 68 patients died. Of these, 43 (75%) died of extracranial disease related to the primary tumor, 10 (18%) died of intracranial lesions due to brain metastases (seven died due to an intracranial mass and three died due to leptomeningeal metastases), and four (7%) died of unknown causes.

### Factors affecting prognosis after diagnosis of brain metastases

According to univariate analysis, sex, timing of brain metastases (synchronous versus metachronous), primary tumor site (colon versus rectum), and brain lesion site (infratentorial versus supratentorial versus both) were not associated with prognosis (*p* = 0.320, 0.405, 0.893, and 0.878, respectively). In contrast, history of systemic chemotherapy before the development of brain metastases was significantly associated with worse OS (*p* = 0.028). Thus, we performed multivariate analyses to control for history of systemic chemotherapy before the development of brain metastases as a covariate, in addition to the five well-known prognostic factors of brain metastases from non-specific primary tumors suggested by RPA and DS-GPA (i.e., age, KPS, presence of extracranial metastases, number of brain metastases, and control of primary tumor).

Of the above-mentioned six factors, multivariate Cox proportional hazards regression models revealed that KPS score ≥ 70 [HR of KPS score < 70, 1.88 (95% CI: 1.02–3.59); *p* = 0.045], number of brain lesions ≤3 [HR of number of brain lesions > 3, 2.04 (95% CI: 1.06–3.97); *p* = 0.033], and no history of systemic chemotherapy before the development of brain metastases [HR of past history of systemic chemotherapy, 2.39 (95% CI: 1.28–4.68); *p* = 0.006] were independent factors associated with a better prognosis (Table 2). Age, presence of extracranial metastases, and control of primary tumor were not prognostic factors.

**Table 2:** Univariate and multivariate analyses of factors affecting survival in patients with brain metastases from colorectal cancer

Variable	Category	Median overall survival (months)	Univariate analysis *p* value	Multivariate analysis		
				Hazard ratio	95% CI	*p* value
Age at the time of brain metastases (years)	< 65	6.1 (3.1–10.5)	0.913	ref		0.963
	≥65	7.3 (4.6–10.9)		1.01	0.57–1.82	
Karnofsky performance status score	≥70	6.8 (2.9–12.1)	0.384	ref		0.045
	< 70	6.8 (4.4–10.5)		1.88	1.02–3.59	
Presence of extracranial metastases	Absent (Brain only)	9.7 (3.2–16.3)	0.507	ref		0.943
	Present (Brain and other sites)	6.7 (3.2–8.8)		1.03	0.43–2.05	
Control of primary tumor	Controlled	6.8 (4.6–9.7)	0.941	ref		0.979
	Uncontrolled	6.1 (1.3–39.6)		1.01	0.44–2.18	
Number of brain metastases	Limited: 1–3	8.8 (6.1–12.1)	0.003	ref		0.033
	Multiple: ≥4	3.1 (1.4–7.3)		2.04	1.06–3.97	
History of systemic chemotherapy before brain metastases	No	10.9 (6.1–25.4)	0.003	ref		0.006
	Yes	6.8 (3.1–7.3)		2.39	1.28–4.68	

Data are presented as median (95% CI) or hazard ratio (95% CI)

*CI* Confidence interval

### Treatments for brain metastases from colorectal cancer

As shown in Table 3, treatments for brain metastases significantly differed between the first half (1997–2005) and second half (2006–2015) of the study period (*p* = 0.004). Notably, whereas WBRT alone was performed in 19 of 36 patients (53%) in the first half, it was performed in only three of 31 patients (10%) in the second half. In contrast, whereas SRT alone was performed in 13 of 32 patients (41%) in the second half, it was performed in only four of 36 patients (11%) in the first half.

**Table 3:** Treatment of brain metastases from colorectal cancer

Initial treatment period	First half 1997–2005	Second half 2006–2015	*p* value
Treatment of brain metastases			
WBRT or WBRT + SRT	21 (58%)	3 (9%)	0.004
SRT	4 (11%)	13 (41%)	
Surgery + SRT	3 (8%)	4 (13%)	
Surgery or Surgery + WBRT	4 (11%)	9 (28%)	
Supportive care	4 (11%)	3 (9%)	
Regimen before diagnosis of brain metastases			
None	13 (36%)	9 (28%)	0.011
Fluoropyrimidine only	9 (25%)	4 (13%)	
Fluoropyrimidine + oxaliplatin	2 (6%)	4 (13%)	
Fluoropyrimidine + irinotecan	6 (17%)	0 (0%)	
Fluoropyrimidine + oxaliplatin + irinotecan	6 (17%)	15 (46%)	
Targeted agents			
Not used	34 (94%)	11 (34%)	<.0001
Used	2 (6%)	21 (66%)	

*WBRT* Whole brain radiotherapy, *SRT* Stereotactic radiotherapy

## Discussion

In this study, we demonstrated that KPS, the number of brain lesions, and history of chemotherapy are independent prognostic factors for OS in patients with brain metastases from CRC. Our finding that KPS is an independent prognostic factor is consistent with what was suggested by DS-GPA for patients with brain metastases from gastrointestinal cancers (i.e., CRC, gastric cancer, esophageal cancer), non-small cell lung cancer, breast cancer, renal cell cancer, and malignant melanoma [10, 11]. Similarly, our finding that the number of brain metastases is a prognostic factor is consistent with the results of another study of brain metastases from gastrointestinal cancers [23]. History of systemic chemotherapy before brain metastases was found to be the strongest prognostic factor (HR: 2.39; *p* = 0.006), and patients with this history had poor survival. Although the presence of extracranial metastases and control of primary tumor were not prognostic factors, the cause of death was systemic progression of primary CRC rather than neurologic death for the majority of patients (75% versus 18%) in this study. Taken together, these results suggest the possibility that patients who have more chemotherapy regimens available to try have opportunities to undergo various regimens against systemic progression and thus can achieve a better prognosis. This is consistent with two studies reporting that patients who underwent less chemotherapy prior to the development of brain metastases from CRC survived longer [24, 25].

WBRT, the standard treatment for brain metastases, is associated with late adverse events, such as leukoencephalopathy, hydrocephalus, and cerebral atrophy, which can lead to cognitive dysfunction in 10 to 20% of mpatients who undergo the procedure [26]. Since many patients now live longer after the diagnosis and treatment of brain metastases, there have been increasing concerns regarding treatment-related toxicities associated with WBRT. In the last few decades, SRT, less neurotoxic than and not as invasive as WBRT, is gaining wide acceptance in clinical settings [27]. In a recent phase III randomized controlled trial (JCOG0504) that compared the effects of salvage stereotactic radiosurgery versus surgery with WBRT in patients with 1–4 brain metastases, the median OS was 15.6 months in both the WBRT arm and salvage stereotactic radiosurgery arm; 16% of patients in the WBRT arm experienced grade 2 to 4 cognitive dysfunction, whereas the rate was only 8% in the salvage stereotactic radiosurgery arm (*p* = 0.048) [28]. The JCOG0504 study concluded that ‘salvage stereotactic radiosurgery is noninferior to WBRT and can be established as a standard therapy for patients with ≤4 brain metastases’ [28]. In the present study, treatments performed for brain metastases significantly differed between the first half (1997–2005) and second half (2006–2015) of the study period (Table 3). Notably, WBRT alone was rarely performed in the second half (3%). In contrast, SRT alone was performed in 41% of patients in the second half, but in only 11% of patients in the first half.

In the present study, 1.2% of all Stage IV patients had synchronous brain metastases, which corresponds to 0.20% (14 of 7047 patients) of the entire patient cohort at the time of CRC diagnosis. These results are similar to those of a recent large population-based study [29], in which roughly 1.0% of all patients with CRC were found to have brain metastases, as well as those reported from the Metropolitan Detroit Cancer Surveillance System [1] and a Dutch series [2]. Thus, our study population is considered representative of the global CRC patient population.

This study has potential limitations. First, biases may exist given the retrospective design. Second, the sample size was relatively small. However, to our knowledge, the number of patients with brain metastases from CRC with sufficient background records, including KPS, was one of the largest reported to date. Third, the study period spanned 1997 to 2015. During this long period, treatment strategies as well as the detectability of brain metastases have dramatically changed. Thus, these data will be difficult to compare with other studies focused on novel approaches. Despite these limitations, our findings warrant further studies in a larger patient series of CRC with brain metastases.

## Conclusion

In addition to KPS, the number of brain lesions and history of chemotherapy were found to be independent prognostic factors for OS in patients with brain metastases from CRC. An awareness of these factors may help gastrointestinal surgeons make appropriate choices in the treatment of these patients.

## Data Availability

The datasets used or analysed during the current study are available from the corresponding author on reasonable request.

## Abbreviations

CI: confidence interval
CRC: colorectal cancer
DS-GPA: diagnosis-specific graded prognostic assessment
HR: hazard ratio
KPS: Karnofsky performance status
OS: overall survival
RPA: recursive partitioning analysis
RTOG: Radiation Therapy Oncology Group
SRI: Score Index for Radiosurgery in Brain Metastases
SRT: stereotactic radiotherapy
WBRT: whole-brain radiotherapy
